# On Boldness in the Treatment of Heart Disease

**Published:** 1904-02-20

**Authors:** 


### ON BOLDNESS IN THE TREATMENT OF HEART DISEASE.

Dr. Alexander Morison,[1] speaking on this subject, suggested that physicians, unlike their surgical brethren, were afraid to make full use of the remedies at their disposal when treating cases of heart disease. He described the symptoms of progressive cardiac failure and their treatment by rest, purgatives, dietetics, and small doses of digitalis, strychnine, and so forth. If these methods yield satisfactory results, as they must do in many instances, well and good ; but if they fail, the physician must not delay, but, like the surgeon when he treats an acute abdominal disease, must adopt bolder measures before the case is allowed to drift into a hopeless condition. First, the vascular system should be unloaded by venesection, 4 to 10 ounces of blood being taken from the arm, the anasarcous limbs should be drained by Southey's tubes or by incisions, and any excess of fluid in chest or abdomen removed. Then the patient having been prepared, provided that the vessels are not hopelessly diseased or the heart tethered by firm extraneous adhesions, the powerful cardiac remedies that are contained in the physicians' armamentarium should be exhibited. Digitalis and strophanthus are the most effective of these remedies. Tincture of digitalis in doses of from 15 to 30 minims every four hours should be given until a definite effect is produced upon the action, force and capacity of the dilated and failing heart. This may require a total administration of from 200 to 300 minims, and the result may be permanent or transient. In the majority of cases the physician succeeds in restoring his patient to comparative health for a time, perhaps for years. This method is not free from danger, it needs skill and carefulness to control the agent, but the alternative, in most cases in which the method is applicable, is to stand aside and watch the patient slowly or suddenly die, or to play with remedies in an ineffective and irrational manner.

[1] West London Medical Journal, January, 1904.

